# Health Literacy of People with Substitutive Private Health Insurance in Germany and Their Assessment of the Health System Performance According to Health Literacy Levels: Results from a Survey

**DOI:** 10.3390/ijerph192416711

**Published:** 2022-12-13

**Authors:** Katharina Achstetter, Julia Köppen, Matthias Haltaufderheide, Philipp Hengel, Miriam Blümel, Reinhard Busse

**Affiliations:** Department of Health Care Management and Berlin Centre for Health Economics Research (BerlinHECOR), Technische Universität Berlin, Straße des 17. Juni 135, 10623 Berlin, Germany

**Keywords:** health literacy, Germany, private health insurance, health system performance assessment, HLS-EU-Q16, social context, access, quality, safety

## Abstract

Health literacy (HL) is a competence to find, understand, appraise, and apply health information and is necessary to maneuver the health system successfully. People with low HL are, e.g., under the risk of poor quality and safety of care. Previous research has shown that low HL is more prevalent among, e.g., people with lower social status, lower educational level, and among the elderly. In Germany, people with substitutive private health insurance (PHI) account for 11% of the population and tend to have a higher level of education and social status, but in-detail assessments of their HL are missing so far. Therefore, this study aimed to investigate the HL of PHI insureds in Germany, and to analyze their assessment of the health system according to their HL level. In 2018, 20,000 PHI insureds were invited to participate in a survey, which contained the HLS-EU-Q16, and items covering patient characteristics and the World Health Organization health systems framework goals (e.g., access, quality, safety, responsiveness). Low HL was found for 46.2% of respondents and was more prevalent, e.g., among men and insureds with a low subjective social status. The health system performance was perceived poorer by respondents with low HL. Future initiatives to strengthen health systems should focus on promoting HL.

## 1. Introduction

Health literacy (HL) describes a central competence and important prerequisite to maneuver the health system successfully, and is essential for individuals to engage and interact with health care providers and institutions [1]. HL can be defined as the ability to find, understand, appraise, and apply health information for everyday health-related decisions within the fields of health care, disease prevention, and health promotion [2]. The concept of HL comprises the knowledge of health, health care and health systems, the use of health-related information, and the skills of maintaining health through both self-management and partnerships with health care providers [3]. HL is an important determinant of health [4] and is associated with health behavior, the use of health care, and health outcomes [2]. People with low HL show fewer positive experiences with patient-centered care and shared decision-making [1] and are under the risk of poor quality and safety of care [2,5]. Therefore, low HL impacts the individuals themselves, but also the health system and society in its entirety, e.g., the individual’s increased use of health care leads to higher health care costs for society [6]. Previous research has identified certain patient characteristics associated with low HL and raises concerns of social inequities. Low HL is more prevalent among, e.g., people with lower social status, lower educational level, and among the elderly [7,8]. However, HL is not only associated with individual patient characteristics, but also shaped by culture and social context factors, such as the country-specific organization and provision of health care [4,9,10]. In addition, HL is influenced by sectors other than health care, such as education and the media [4]. This emphasizes the importance of addressing the social context, such as health insurance schemes, interactions with health care providers, health care organizations, and the overall health system, when measuring HL.

Health insurance is mandatory in Germany and the health insurance system consists of statutory (SHI) and private health insurance (PHI). Whereas most people are covered by SHI, substitutive PHI accounts for 11% of the population. Employees, whose income is above a fixed threshold (in 2022: EUR 64,350 gross annual income), the self-employed, and civil servants, can or must opt to enroll in PHI. In general, PHI insureds use the same health care services and are in contact with the same providers as SHI insureds in Germany. PHI insureds are required to pay (ambulatory) providers directly based on a price list for privately delivered medical services. Afterwards, they can receive reimbursement from their insurance company [11]. PHI insureds tend to be younger, healthier, have a higher educational level, and a higher social status, compared to SHI insureds [12,13,14,15]. Accordingly, it can be hypothesized that low HL is less prevalent among people with PHI. The prevalence of low HL was found to be between 44–46% for the general population in Germany, with some studies even reporting low HL for 54% of the German population [8,16]. However, a representative survey in Germany comprising a small sample of PHI insureds (*n* = 136) found a slightly higher prevalence of low HL in PHI compared to SHI (57.0% vs. 54.1%) [8]. To date, little research has focused on the HL of German PHI insureds. Research lacks on how the social context of PHI insureds, i.e., their health insurance scheme and their interaction with the health system, shapes their HL levels. Furthermore, it can be hypothesized that variations of HL by subgroups according to the well-researched associated characteristics, such as age, educational level, and subjective social status, can also be found among PHI insureds. Hence, identifying subgroups with low HL levels would be beneficial in understanding which population groups might be most vulnerable in this specific population group.

Since previous research indicated that low HL is associated with low satisfaction with health care [17,18,19], and poor quality and safety of care [1], it can be assumed that people with low HL also have poorer perceptions of other aspects of care. Therefore, it can be hypothesized that the PHI insureds’ assessment of the health system varies according to HL levels. Health system performance assessment (HSPA) is a tool to measure, monitor, and evaluate the performance of a specific health system along predefined goals [20]. The World Health Organization (WHO) (2007) defined in its health systems framework the intermediate and final goals of high-performing health systems. The intermediate goals are access, coverage, quality, and safety, and the final goals are improved health, responsiveness, social and financial risk protection, and improved efficiency [21,22]. Assessing the performance of a health system helps to identify weaknesses of the system, aims to identify inequities between certain population groups, and is a prerequisite for strategies to strengthen the health system and for promoting patient-centered care. Integrating the population perspective into health care evaluation and performance assessment has become highly relevant [21,23,24]. 

PHI insureds in Germany are said to be often overtreated and prioritized by health care providers, e.g., due to financial incentives for physicians who can charge higher fees for the same services, than for SHI insureds [25]. However, PHI insureds are strongly underrepresented in health services research in Germany and their perception of the health system performance was mostly unclear. Therefore, we conducted a comprehensive HSPA from their perspective along the WHO’s goals (project IPHA “Integrating the Population Perspective in Health System Performance Assessment”, see [26]). This revealed insights into the overall health system performance from the PHI insureds’ perspective, and further uncovered performance variations and inequities between sociodemographic subgroups (e.g., regarding age, gender, income, health status) [27]. As the performance was not homogenously perceived by this specific population group, it was further assumed that other patient characteristics, such as health literacy levels, might also uncover inequities. Hence, it was hypothesized that the HSPA differs by HL levels of PHI insureds. Investigating the health system performance according to HL levels might help reveal areas of concern, such as access barriers or perceived discrimination, for people with low HL. In the future, this allows to broaden the understanding of differences in the perception of the health system performance, to capture resulting challenges for health care, and to derive interventions for improving health care, and strengthening the health system. 

This study aims: (1) to investigate the HL of people with PHI in Germany; (2) to identify differences in HL levels among subgroups (e.g., age, educational level, social status); and (3) to analyze differences in the assessment of the health system (e.g., regarding access, quality, safety, responsiveness) according to HL levels.

## 2. Materials and Methods

Between October and December 2018, 20,000 people with substitutive private health insurance from ‘Debeka’ were invited to participate in a quantitative cross-sectional survey [26,27]. The study was approved by the Ethics Committee of the Charité – Universitätsmedizin Berlin (EA4/075/18).

### 2.1. Sample

A random sample of PHI insureds enrolled with the provider Debeka (one of the largest PHI companies in Germany) was drawn, stratified by gender, age, and aid allowance (for civil servants) according to all PHI insureds in Germany to aim for representativeness [28,29]. Additionally, an over-recruitment of 10% in the 18–34 age group and an under-recruitment of 10% in the 65 + age group were planned based on the previous experiences [30]. All persons aged 18 years and older who had been continuously insured with Debeka since January 2015 were included in the sample. People with long term care grades 4 or 5 and at the end of life (in a hospice) were excluded. 

Respondents were contacted once by Debeka with a cover letter containing the study description, a consent statement, a postage-free return envelope, and a paper questionnaire and were invited to participate. The cover letter also provided a link to the online questionnaire (SoSci Survey), and participants had free choice between a paper- and a web-based survey. All materials were provided in German language and no incentives were offered for participation.

### 2.2. Survey Items

Survey items included sociodemographic variables (e.g., age, gender, education), patient characteristics (e.g., HL, health status), and items assessing the health system performance following the intermediate and final goals of the WHO health systems framework: access, coverage, quality, safety, improved health, responsiveness, social and financial risk protection, and improved efficiency.

The questionnaire used primarily validated survey items (e.g., which are nationally/internationally established in surveys conducted by the Commonwealth Fund, Eurostat, Robert Koch Institute, the WHO). In case of the unavailability of validated survey items, adapted or newly developed questions were included (e.g., the perception of quality differences between hospitals, the perception of the health insurance premium in relation to the coverage) (see study protocol for details [26]). HL was assessed with the German version of the HLS-EU-Q16. Respondents were asked by using the 16 HL items and a four-point Likert scale: ‘very difficult’, ‘fairly difficult’, ‘fairly easy’, ‘very easy’, and ‘don’t know’ [31]. 

The questionnaire was pretested (*n* = 122) for content and comprehensibility, the web-based survey (mobile and desktop versions) was additionally tested for technical feasibility, and both were modified accordingly.

### 2.3. Analysis

The paper questionnaires were processed electronically and merged with the web-based data subsequently. Frequencies, means, standard deviation, and confidence intervals were calculated. Differences between subgroups were analyzed by chi square test, t-test (for mean age), or Mann-Whitney U test (for visual analogue scale) with a significance level of *p* < 0.05 using IBM SPSS Statistics 27 (IBM, New York, NY, USA).

### 2.4. Health Literacy Score

The HL score was calculated following the manual for the HLS-EU-Q16 [32]. Respondents had to answer at least 14 of the 16 questions of the HLS-EU-Q16 for generating a HL score. Responses of ‘very easy’ or ‘fairly easy’ were coded as 1, the categories ‘fairly difficult’ or ‘very difficult’ as 0 and a HL sum score was calculated. ‘Don’t know’ and omitted items were treated as missing values. A sum score of 0 to 8 was considered inadequate, a score between 9 and 12 was considered problematic, and a score of 13 or more was considered sufficient HL. For further analyses, HL levels were dichotomized into low (inadequate/problematic), and high (sufficient) HL as suggested by Dahlman et al. (2020) [33].

## 3. Results

Of the 20,000 invited PHI insureds, a total of *n* = 3617 individuals participated in the survey (3307 paper-based, 310 web-based). After plausibility checks and data cleansing, 16 persons were excluded, e.g., due to double participation, or empty questionnaire, and finally, the answers of 3601 participants (18.0% response rate) were available. 

### 3.1. Sample Characteristics

The total sample (*n* = 3601) had a mean age of 58.5 years (±14.6) and 64.7% of the respondents were male (see Table 1). A high educational level (ISCED 5–8) was found for 80.5% of the total sample. Most respondents worked full-time (44.3%) or were retired (40.2%). The majority of respondents (71.9%) rated their health as very good or good, but still 58.9% reported one or more chronic diseases.

Of the total 3601 respondents, 22.2% had missing values or answered ‘don’t know’ for more than two of the 16 items of the HLS-EU-Q16 questionnaire. Thus, it was possible to calculate a HL score for 2801 respondents. 

The availability of a HL score among respondents differed according to sociodemographic characteristics (see Table 1). For respondents for whom a HL score could be calculated, the average age was 57.8 years (±14.3) and 81.3% had a high educational level. Respondents without a HL score were significantly more often in the age group 75+, had a low or medium educational level (ISCED 0–4), a lower income, less often chronic diseases, and were less often taking (very) much care of their health. No gender differences were seen.

Furthermore, the availability of a HL score was higher among the participants using the web-based survey (83.1% vs. 77.3%). In addition, the sample differed significantly regarding paper-based or web-based participation. Respondents participating online were more often male, between 18 and 64 years, in full-time employment, with a very good health status, and without chronic diseases.

### 3.2. Results of the HLS-EU-Q16 Items

**Figure 1:**
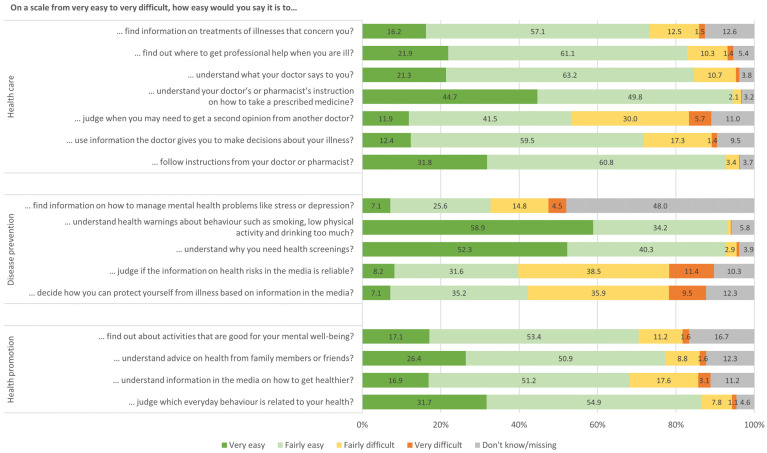
Percentage distribution of responses for the HLS-EU-Q16 items sorted by fields of health care, disease prevention and health promotion (*n* = 3601). Note: values <1.0% are not shown.

Response rates varied for each of the HLS-EU-Q16 items, resulting in a range of missing values and ‘don’t know’ answers between 3.2% and 16.7%, and were 48.0% for one item (see Figure 1). Most respondents stated that it is very/fairly easy to understand instructions from physicians/pharmacists on how to take prescribed medicines (94.5%); to understand health warnings about risky health behavior (93.1%); to understand the reasons for health screenings (92.6%); and to follow instructions from a physician/pharmacist (92.6%). In total, 13 of 16 items were rated as very/fairly easy by more than half of the respondents (ranging between 53.4% and 94.5%). In contrast, only 32.7% of the respondents stated that it is very/fairly easy to find information regarding mental health problems (see Figure 1). Besides, two other items in the area of “disease prevention” were only rated as very/fairly easy by 39.8% (judge the reliability of media information on health risks) and 42.3% (deciding how to protect oneself from illness based on media information).

### 3.3. Health Literacy Levels and Subgroup Differences by Socioeconomic and Health Characteristics

For all analyses using HL levels, only respondents with a valid HL score were included (*n* = 2801). Low HL was found for 46.2% (9.4% inadequate and 36.8% problematic) of respondents and high (sufficient) HL for 53.8% (see Table 2). 

Subgroup differences (see Table 3) according to HL levels can be seen for several sociodemographic characteristics. Low HL (inadequate/problematic) was more often observed among men, in the age groups 18 to 49 years, among those working full-time, with a low subjective social status, with a net equivalent income up to EUR 1136 (at risk of poverty threshold), with a very bad or bad self-rated health status, and among those less/not at all taking care of their health. The highest share of respondents with low HL was found for respondents with (very) bad health status (61.3%), in the lower middle class (60.2%), and for the lowest income group (59.7%). Differences regarding the educational level were not significant (low HL ranging between 41.0% and 47.2% of respondents), which might be due to the low number of people with low educational level.

### 3.4. Health System Performance Assessment according to Health Literacy Levels

The assessment of health system performance along the WHO health systems framework goals from the PHI insureds’ perspective, stratified by HL levels, can be seen in Table 4. The results show statistically significant differences between respondents with low and high HL for all areas, with the most obvious differences for the following: people with low HL were less often (very) satisfied with the German health system in comparison to respondents with high HL (60.0% vs. 73.2%). Furthermore, respondents with low HL reported more often difficulties when accessing after-hours medical care (64.9% vs. 49.8%), unmet needs due to waiting time, distance, or financial reasons, and the use of health services which needed to be paid out-of-pocket (OOP). Besides, respondents with low HL perceived more often notable quality differences between hospitals (81.1% vs. 72.6%), discrimination experiences in the past year (12.6% vs. 4.5%), high needs for reforms in Germany for all eight areas and reported safety concerns such as receiving wrong medication (10.1% vs. 5.1%), suspecting medical errors (20.2% vs. 11.8%), and receiving wrong test results (6.3% vs. 2.9%). Furthermore, respondents with low HL perceived more often strong financial burden by OOP spending (15.1% vs. 8.1%), difficulties with paying the health insurance premiums (3.6% vs. 2.0%), inefficiencies such as duplicate tests, and unnecessary health care services, and perceived the relation of insurance premiums to coverage as (too) high (35.6% vs. 31.3%). Additionally, respondents with low HL rated the nine responsiveness items concerning the last physicians visit less often as (very) good, e.g., for coordination of care among different physicians (46.1% vs. 71.1%) or participation in shared decision-making (78.4% vs. 93.5%).

## 4. Discussion

This study aimed to measure the HL of PHI insureds in Germany, finding low HL for 46.2%, and highlighted subgroup differences, e.g., low HL was found more frequently in participants aged 18 to 49, with low educational level, low income, and low social status. Furthermore, differences in the assessment of the health system performance (e.g., regarding access, quality, safety) according to HL levels were identified, resulting in a lower performance of the German health system for people with low HL. 

This study focuses on German PHI insureds who are not well-researched yet, and provides novel findings and valuable insights into this population group. The total sample had a high average age (58.6 years), 58.9% of respondents reported chronic diseases, 80.5% had a high educational level (ISCED 5–8), and 86.6% had a net equivalent income above the median of the German population. In this respect, our findings contrast with the assumption that PHI insureds are younger and healthier than the overall German population [12,13,14,15]. In addition, the extraordinary high educational level and income characterize the respondents as a specific population group and emphasize this particular social context. The link between education and PHI insurance was already found in a previous survey (2012), where PHI insureds had higher educational levels compared to SHI insureds [15]. 

Due to the innovation of this study and lack of previous research on the HL levels of PHI insureds in Germany, the results of this study need to be compared to research of the total population. The PHI insureds’ rating of the HLS-EU-Q16 items varied greatly: understanding a physician’s or pharmacist’s instruction on how to take prescribed medicine was rated as very/fairly difficult by 2.3% of the respondents. This is in line with Jordan and Hoebel (2015) who identified 4.0% in a German survey of the general population in 2013/14 [31]. Finding information on mental health problems, such as stress or depression, was rated as very/fairly difficult by 19.3% of the respondents in our study, but another 48.0% stated ‘don’t know’ or omitted the question. In contrast, Jordan and Hoebel found that 36.9% perceived it very/fairly difficult [31]. This shows that finding information on the management of mental health problems may remain difficult for all population groups and knowledge about mental health services do not seem to be very present for people not in need, resulting in many ‘don’t know’ answers. Judging the reliability of health risk information from the media was perceived to be very/fairly difficult by 49.9% of respondents, which is comparable to the results of the survey by Jordan and Hoebel (2015) at 50.7% [31]. Assumingly, the reliability of media information has not significantly improved over time and there seems to be a high need for reliable information sources and for education programs advancing media competencies in Germany. The field of “disease prevention” contains three items with the highest numbers of very/fairly difficult answers. This implies that knowledge on the prevention of diseases should be further promoted and supported by targeted interventions. Overall, the HLS-EU-Q16 items showed a wide variation of the number of missing values (3.2−48.0%). In total, 22.2% of the respondents did not answer at least 14 of the 16 items, which would have been necessary for a valid HL score. In a previous study by Rademakers and Heijmans (2018), 67.0% of the sample had a valid HL score and 30.7% had a high educational level [1]. This contrasts with our study, where 77.8% of respondents had a valid HL score and 80.5% a high educational level. Hence, it can be assumed that the high educational level of our sample was one reason for the comparatively lower number of missing values. It is noteworthy that 50.0% of those who did not have a valid HL score, stated to have no chronic disease. It can be assumed that especially people with less contact with the health system cannot assess health information for all given examples in the HLS-EU-Q16 in the fields of health care, disease prevention and health promotion. Thus, respondents without a valid HL score differ significantly from people with a HL score. This is partly supported by research from Ehmann et al. (2020), who found that participants with and without a valid HLS-EU-Q16 score differed regarding health status, but not regarding age, years of school education, and employment status [35]. Further research should investigate these associations and their influence on the measurement of HL. In addition, discussing and enhancing the tool with the aim of reaching higher response rates and including people with fewer health care experiences should be on the agenda of HL experts. 

Of those respondents with a valid HL score, almost half (46.2%) showed low HL. Despite the PHI insureds’ specific social context, i.e., their health insurance scheme, their interactions with health care providers and institutions, and their specific characteristics, i.e., their high educational level and subjective social status, the share of people with low HL does not differ from the total population (44–46%) [16]. This leads to the assumption that PHI insurance as a social context factor has no strong influence on HL per se, but the associated characteristics (e.g., subjective social status, health status) have. Low HL was more often found among men, respondents aged 18–49, full-time workers, with low subjective social status, low income, bad health status, with chronic diseases, and not taking very much care of one’s own health. HL is a particularly strong predictor of the health status [36]. This is supported by our findings, as the highest share of respondents with low HL (61.3%) was found for respondents with (very) bad health status. Levin-Zamir et al. (2017) found education and income to be strongly associated with HL in a survey among adults in Israel [37]. This finding is only partly supported by our results, with 59.7% respondents having low HL in the lowest income group compared to 42.7% having low HL in the highest income group. However, we found no significant differences for education, which might be due to low variance in the extraordinary high educational level of our study sample. Schaeffer et al. (2017) found the elderly (65 + years) and people with low subjective social status to be more likely to have low HL levels [8]. This finding is partly in line with our results, with more respondents with a low subjective social status having low HL. However, this study found more younger people having low HL, which might be due to a lack of experiences with health care, particularly in the age group 18–34. The finding that the working-age population group (especially those between 18 and 49) has a high proportion with low HL is particularly critical in the context of the results of Eichler et al. (2009), who addressed the financial costs of limited HL [38]. This is of high relevance in respect to the increasing health care cost and the economic burden on society. Overall, these findings might be influenced by our focus on PHI insureds and their particular sociodemographic characteristics, i.e., high educational level, high subjective social status, and high income. Future research could examine the extent of the associations between patient characteristics and HL among PHI insureds and explore their differences from those in the general population.

The assessment of the health system performance varied strongly between respondents with high and low HL. Satisfaction with the health system was lower among people with low HL. Altin and Stock (2016) also found this association among a sample of the German population [19]. This leads to the assumption that HL and satisfaction are highly connected, and that the link between these should be further investigated, to understand if strengthening HL might also improve overall satisfaction with health care. Furthermore, respondents with low HL more often perceived access barriers, unmet needs, more OOPs, quality differences, safety concerns in their care, and discrimination. Responsiveness was rated worse, more needs for reforms were stated, respondents were more financially burdened, and inefficiencies were reported more often by respondents with low HL. This indicates that respondents with low HL form a vulnerable population group for experiencing low health system performance and the health system does not meet their expectations. Furthermore, it needs to be clarified if people with more than one vulnerable characteristic, e.g., the combination of low HL and low subjective status, are under a higher risk of perceiving and also receiving poor health care. 

Our study findings are supported by Levy and Janke, (2016), who found that access to health care was more difficult for people with low HL [39]. The accessibility of health care services requires knowledge, which might be limited among people with low HL. Therefore, it can be concluded that strengthening HL might also help to improve the access to health care. Respondents with low HL perceived more often notable quality differences between hospitals, but had less often knowledge of hospital quality reports. Hospitals are legally obliged to publish quality reports in Germany with the intention to provide a trustworthy information source for patients which can be used for choosing a hospital [11,40]. However, this information source does not seem to be widely accepted and/or known by people with low HL. It needs to be further explored if this information source is too complex or too hard to find for people with low HL, or if it just needs more advertisement. Basically, it can be recommended that quality reports should be prepared in a patient-oriented way in the future and that they should be easier to find. Safety risks were already shown to be associated with low HL in previous research [1]. This is in line with our study, where respondents with low HL experienced safety concerns significantly more often during the provision of health care. Discrimination experiences were also found to be associated with low HL by Lyles et al. (2011) [41]. Likewise, our study revealed more discrimination experiences by respondents with low HL. This can be seen as counterfactual that PHI insureds are prioritized by health care providers, when even a significant share of this per se privileged population group perceives discrimination in their care. Moreover, the financial advantages for physicians when treating PHI insureds, and the profit motivated provision of more and sometimes even unnecessary services might lead to an atypical form of discrimination [11,25]. Perceiving the provision of unnecessary services was reported by both respondents with low and high HL, ranging between every fifth and every third respondent. This could be explored in future research with the aim of minimizing discrimination experiences for all patients. Coordination between different physicians was perceived as (very) good by only 46.1% of respondents with low HL. This indicates a high need for improving the coordination of care in Germany, particularly for people with low HL. Individuals must have broad competencies to compensate for the lack of coordination between physicians. It can be further assumed that these competencies are limited among people with low HL, so they might tend to have a higher need for well-working coordination structures between providers. The knowledge and information asymmetry between health care providers and patients, in particular with low HL [6], might be a further hurdle when assessing, e.g., the coordination between providers. Furthermore, it remains unclear, if people with low HL feel stronger burdened by their OOP spending due to an increased use of health care or due to its link with the social status. This might be subject to further research.

Due to the specific characteristics of PHI insureds, we sought to broaden the insights into their perception of health care and the health system. We gained insights into the link between their specific social context and HL. We further highlighted the health system performance disparities between people with low and high HL. Consequently, strengthening HL and being more responsive to individual characteristics, needs, and expectations, should be a major goal of health care providers and should be also included in future health research and policies. 

## 5. Limitations

As a major strength, this study provides novel insight into the HL of PHI insureds and shows differences in their assessment of the performance of the German health care system according to HL levels. However, some limitations have to be considered when interpreting the results. Although the sample was stratified by age, gender, and aid allowance according to all PHI insureds in Germany, our final sample differed slightly from the drawn sample. Furthermore, the study sample only consists of individuals from one PHI company and therefore the results might not be representative for all PHI insureds in Germany. This needs to be considered when interpreting the results. As previous research identified differences between various sickness funds, e.g., due to regional variations, this might also be applicable for PHI companies [13]. In addition, the questionnaire was provided only in German, which excluded respondents with insufficient knowledge of the German language. Furthermore, individuals who had little experience with the German health care system and/or health care were disadvantaged in answering very specific questions, e.g., regarding mental health problems or the coordination of care between physicians. The HLS-EU-Q16 is a validated and widely used tool for measuring self-reported HL. However, it is a short form of the HLS-EU-Q47 and thus only captures a partial picture of competencies. Furthermore, it can be assumed that the self-reported measurement of HL is affected by unmeasured factors. For example, it might be possible that PHI insureds with a high educational level have a better perception of the complexity of the German health care system and therefore rate their own HL lower. Hence, an objectively measurement HL might reveal higher HL levels compared to the total population. This could be subject to further research. 

## 6. Conclusions

Promoting HL in Germany in a systematic manner should be high on the agenda of policymakers and requires a health agenda in all policies strategy. As the health system performance was perceived poorer by respondents with low HL, promoting HL can be understood as a goal of high-performing health systems. Hence, health systems should particularly focus their strategies and policy measures on the needs of people perceiving the lowest health system performance. Furthermore, efforts for strengthening the health system and promoting equity need to address and promote HL. In addition, promoting HL should be pursued early on, in different settings, and independent of sociodemographic characteristics. The “education for health literacy” approach aims at promoting HL in childhood and adolescence through health education in schools and, hence, sets the cornerstone for HL during adulthood [42]. This approach could be considered as one main strategy for promoting HL in Germany.

Incorporating the social context, e.g., interactions with the health system or the health insurance scheme, and simultaneously addressing individual characteristics, e.g., sociodemographic characteristics, and previous experiences with health care, should be included in the future when measuring and discussing HL. Besides, these characteristics are essential when developing tailored strategies for improving HL in the population. 

Empowering patients to maneuver the health system with confidence requires a solid HL level, but more importantly, health literate health care providers, institutions, organizations — overall, a health literate health system. As the German health care system is highly fragmented and particularly complex, it puts the patients under high demands to navigate through the system. Therefore, the concept of “navigation health literacy” should be further promoted in Germany. Navigation health literacy encompasses competencies allowing patients to navigate the system successfully and without difficulties, but also addresses the complexity of the health system itself [43]. Additionally, the concept of “health literate systems” [44] should be further explored and strengthened in Germany. Health literate systems focus both on creating enabling environments and supporting patients’ HL [44]. The HL of health systems is closely linked to the HL of health professionals. They need to be health literate for their own professional decisions and actions, but also for supporting patients and strengthening their HL, decision-making, and health-related behaviors. The importance of this was already shown by previous research [45]. Therefore, it is of high relevance to integrate measures aiming at strengthening the HL of health professionals, and thereby to promote health literate systems into health policies on a national level. 

## Figures and Tables

**Table 1:** Sample characteristics, total and stratified by availability of health literacy score (*n* = 3601).

			Health Literacy Score Available ^a^			
	Total		Yes		No	
	*n*	%	*n*	%	*n*	%
Total	3601	100	2801	77.8	800	22.2
Gender (*n* = 3589)						
Male	2323	64.7	1806	64.7	517	64.9
Female	1266	35.3	986	35.3	280	35.1
Age (years) (*n* = 3588)						
Mean (SD) ^b^	58.5 (14.6)		**57.8 (14.3)**		**60.0 (15.6)**	
18–34	307	8.6	**243**	**8.7**	**64**	**8.0**
35–49	637	17.8	**509**	**18.2**	**128**	**16.1**
50–64	1220	34.0	**999**	**35.8**	**221**	**27.8**
65–74	928	25.9	**717**	**25.7**	**211**	**26.5**
75+	496	13.8	**324**	**11.6**	**172**	**21.6**
Educational level (*n* = 3572)						
Low (ISCED 0–1)	57	1.6	**39**	**1.4**	**18**	**2.3**
Medium (ISCED 2–4)	641	17.9	**481**	**17.3**	**160**	**20.3**
High (ISCED 5–8)	2874	80.5	**2264**	**81.3**	**610**	**77.4**
Work status (*n* = 3560)						
Full-time	1578	44.3	**1274**	**45.8**	**304**	**39.1**
Part-time	315	8.8	**257**	**9.2**	**58**	**7.5**
Retired	1431	40.2	**1068**	**38.4**	**363**	**46.7**
Other (student, parental leave, unemployed, etc.)	236	6.6	**183**	**6.6**	**53**	**6.8**
Monthly net equivalent income ^c^ (*n* = 3399)						
Up to €1136	81	2.4	**62**	**2.3**	**19**	**2.6**
€1137–€1893	374	11.0	**277**	**10.4**	**97**	**13.3**
€1894–€2839	1418	41.7	**1084**	**40.6**	**334**	**45.7**
€2840+	1526	44.9	**1245**	**46.7**	**281**	**38.4**
Subjective social status (*n* = 3514)						
Lower middle class	150	4.3	**113**	**4.1**	**37**	**4.9**
Middle class	1616	46.0	**1247**	**45.3**	**369**	**48.6**
Upper middle class	1512	43.0	**1222**	**44.4**	**290**	**38.2**
Upper class	137	3.9	**103**	**3.7**	**34**	**4.5**
None of those	99	2.8	**69**	**2.5**	**30**	**3.9**
Self-rated health (*n* = 3582)						
(Very) bad	119	3.3	**93**	**3.3**	**26**	**3.3**
Moderate	890	24.8	**710**	**25.5**	**180**	**22.7**
Good	2098	58.6	**1646**	**59.0**	**452**	**56.9**
Very good	475	13.3	**339**	**12.2**	**136**	**17.1**
Chronic diseases (*n* = 3584)						
None	1475	41.2	**1079**	**38.6**	**396**	**50.0**
Yes, one	1247	34.8	**998**	**35.7**	**249**	**31.4**
Yes, several	862	24.1	**715**	**25.6**	**147**	**18.6**
Taking care of own health (*n* = 3589)						
Less/not at all	93	2.6	**56**	**2.0**	**37**	**4.7**
Moderate	1213	33.8	**912**	**32.6**	**301**	**38.0**
(Very) much	2283	63.6	**1829**	**65.4**	**454**	**57.3**

Abbreviations: SD—standard deviation; ISCED — International Standard Classification of Education. Notes: ^a^ health literacy score was calculated in the case of at least 14 of 16 valid answers; ^b^ *t*-test was used for mean age; ^c^ groups were built in relation to the monthly net equivalent income of the German population in 2018 (€1894): <60%, <100%, <150%, and ≥150% [34]; bold values indicate a statistically significant difference with a *p*-value <0.05 (chi square test); percentages may not total 100 due to rounding.

**Table 2:** Health literacy levels (*n* = 2801).

Health Literacy Levels	*n*	%	95%-CI
Inadequate (score 0–8)	263	9.4	8.4–10.5
Problematic (score 9–12)	1032	36.8	35.1–38.6
Sufficient (score 13–16)	1506	53.8	51.9–55.6

Abbreviation: CI—confidence interval.

**Table 3:** Sample characteristics stratified by health literacy levels (*n* = 2801).

	Health Literacy Level				
	Low (Inadequate/Problematic)		High (Sufficient)		
	*n*	%	*n*	%	*p*-Value
Total	1295	46.2	1506	53.8	
Gender (*n* = 2792)					**0.023**
Male	**863**	**47.8**	**943**	**52.2**	
Female	**427**	**43.3**	**559**	**56.7**	
Age (years) (*n* = 2792)					**<0.001**
18–34	**131**	**53.9**	**112**	**46.1**	
35–49	**269**	**52.8**	**240**	**47.2**	
50–64	**482**	**48.2**	**517**	**51.8**	
65–74	**275**	**38.4**	**442**	**61.6**	
75+	**132**	**40.7**	**192**	**59.3**	
Educational level (*n* = 2784)					0.735
Low (ISCED 0–1)	16	41.0	23	59.0	
Medium (ISCED 2–4)	227	47.2	254	52.8	
High (ISCED 5–8)	1044	46.1	1220	53.9	
Work status (*n* = 2782)					**<0.001**
Full-time	**644**	**50.5**	**630**	**49.5**	
Part-time	**117**	**45.5**	**140**	**54.5**	
Retired	**434**	**40.6**	**634**	**59.4**	
Other (student, parental leave, unemployed, etc.)	**88**	**48.1**	**95**	**51.9**	
Monthly net equivalent income ^a^ (*n* = 2668)					**<0.001**
Up to €1136	**37**	**59.7**	**25**	**40.3**	
€1137–€1893	**154**	**55.6**	**123**	**44.4**	
€1894–€2839	**505**	**46.6**	**579**	**53.4**	
€2840+	**532**	**42.7**	**713**	**57.3**	
Subjective social status (*n* = 2754)					**<0.001**
Lower middle class	**68**	**60.2**	**45**	**39.8**	
Middle class	**622**	**49.9**	**625**	**50.1**	
Upper middle class	**514**	**42.1**	**708**	**57.9**	
Upper class	**40**	**38.8**	**63**	**61.2**	
None of those	**30**	**43.5**	**39**	**56.5**	
Self-rated health (*n* = 2788)					**<0.001**
(Very) bad	**57**	**61.3**	**36**	**38.7**	
Moderate	**366**	**51.5**	**344**	**48.5**	
Good	**747**	**45.4**	**899**	**54.6**	
Very good	**121**	**35.7**	**218**	**64.3**	
Chronic diseases (*n* = 2792)					**0.040**
None	**473**	**43.8**	**606**	**56.2**	
Yes, one	**460**	**46.1**	**538**	**53.9**	
Yes, several	**357**	**49.9**	**358**	**50.1**	
Taking care of own health (*n* = 2797)					**0.001**
Less/not at all	**33**	**58.9**	**23**	**41.1**	
Moderate	**458**	**50.2**	**454**	**49.8**	
(Very) much	**802**	**43.8**	**1027**	**56.2**	

Abbreviations: ISCED—International Standard Classification of Education; SHI—Statutory Health Insurance. Notes: ^a^ groups were built in relation to the monthly net equivalent income of the German population in 2018 (€1894): <60%, <100%, <150%, and ≥150% [34]; bold values indicate a statistically significant difference with a *p*-value <0.05 (chi square test); percentages may not total 100 due to rounding.

**Table 4:** Assessment of the health system performance from the PHI insureds’ perspective, total and stratified by health literacy levels (*n* = 2801).

			Health Literacy Level				
	Total *n* = 2801		Low (Inadequate/Problematic) *n* = 1295		High (Sufficient) *n* = 1506		
	*n*	%	*n*	% [95%-CI]	*n*	% [95%-CI]	*p*-Value
Satisfaction							
Overall satisfaction with the health system (*n* = 2718)							**<0.001**
(Very) dissatisfied	127	4.7	**81**	**6.4 [5.2–7.9]**	**46**	**3.2 [2.4–4.2]**	
Neither satisfied nor dissatisfied	768	28.3	**423**	**33.5 [31.0–36.2]**	**345**	**23.7 [21.5–25.9]**	
(Very) satisfied	1823	67.1	**757**	**60.0 [57.3–62.7]**	**1066**	**73.2 [70.8–75.4]**	
Access							
Accessing after-hours medical care (*n* = 2796)							**<0.001**
Very/somewhat difficult	1588	56.8	**839**	**64.9 [62.3–67.5]**	**749**	**49.8 [47.3–52.3]**	
Very/somewhat easy	593	21.2	**208**	**16.1 [14.2–18.2]**	**385**	**25.6 [23.4–27.8]**	
Never needed after-hours medical care	615	22.0	**245**	**19.0 [16.9–21.2]**	**370**	**24.6 [22.5–26.8]**	
Unmet needs due to ^a^							
Waiting time (*n* = 2580)	207	8.0	**136**	**11.5 [9.8–13.4]**	**71**	**5.1 [4.0–6.3]**	**<0.001**
Distance (*n* = 2551)	90	3.5	**61**	**5.2 [4.1–6.6]**	**29**	**2.1 [1.4–3.0]**	**<0.001**
Financial reasons (*n* = 2622)	199	7.6	**135**	**11.0 [9.4–12.9]**	**64**	**4.6 [3.6–5.8]**	**<0.001**
Coverage							
Out-of-pocket health spending in the past year ^b^ (*n* = 2671)							
Medical products (pharmaceuti-cals and medical aids)	2043	76.5	**976**	**78.7 [76.4–80.9]**	**1067**	**74.6 [72.3–76.8]**	**0.012**
Deductible	1202	45.0	**603**	**48.6 [45.9–51.4]**	**599**	**41.9 [39.3–44.4]**	**<0.001**
Services (inpatient/ambulatory care by physicians/allied health professionals)	659	24.7	**351**	**28.3 [25.9–30.9]**	**308**	**21.5 [19.5–23.7]**	**<0.001**
Dental care	661	24.7	**354**	**28.5 [26.1–31.1]**	**307**	**21.5 [19.4–23.6]**	**<0.001**
Other services	529	19.8	**275**	**22.2 [19.9–24.6]**	**254**	**17.7 [15.8–19.8]**	**0.004**
No out-of-pocket spending	351	13.1	**131**	**10.4 [8.8–12.2]**	**220**	**15.2 [13.4–17.1]**	**<0.001**
Quality							
Reasons for hospital choice ^b^ (*n* = 2562)							
Reputation	2028	79,2	922	78.0 [75.6–80.3]	1106	80.1 [78.0–82.2]	0.183
Medical quality	1596	62.3	739	62.5 [59.7–65.2]	857	62.1 [59.5–64.6]	0.827
Amenities	583	22.8	**291**	**24.6 [22.2–27.1]**	**292**	**21.2 [19.1–23.4]**	**0.037**
Quality differences between hospitals (*n* = 2746)							**<0.001**
No/some differences	645	23.5	**240**	**18.9 [16.8–21.2]**	**405**	**27.4 [25.2–29.7]**	
Notable differences	2101	76.5	**1028**	**81.1 [78.8–83.2]**	**1073**	**72.6 [70.3–74.8]**	
Knowledge about information sources regarding hospital quality ^a^							
Hospital websites (*n* = 2758)	2350	85.2	1088	85.1 [83.0–86.9]	1262	85.3 [83.5–87.1]	0.847
Hospital quality reports (*n* = 2702)	1216	45.0	**497**	**39.4 [36.7–42.1]**	**719**	**49.9 [47.3–52.5]**	**<0.001**
Other sources (*n* = 2730)	1541	56.4	709	55.8 [53.0–58.5]	832	57.0 [54.5–59.5]	0.514
Safety							
Experiences in the past two years ^a^							
Received wrong medication/dose (*n* = 2533)	188	7.4	**118**	**10.1 [8.5–11.9]**	**70**	**5.1 [4.0–6.4]**	**<0.001**
Suspected medical error in treat-ment/care (*n* = 2525)	396	15.7	**234**	**20.2 [18.0–22.6]**	**162**	**11.8 [10.2–13.6]**	**<0.001**
Was told that a medical error had been made (*n* = 2623)	123	4.7	57	4.6 [3.6–5.9]	66	4.7 [3.7–5.9]	0.914
Received wrong results of medical/laboratory tests (*n* = 2400)	107	4.5	**69**	**6.3 [5.0–7.9]**	**38**	**2.9 [2.1–3.9]**	**<0.001**
Improved health							
Perceived health (VAS from 0 [worst] to 100 [best health]), mean (95%-CI) ^c^ (*n* = 2788)	75.7		**1291**	**73.9 [73.0–74.7]**	**1497**	**77.3 [76.6–78.1]**	**<0.001**
Responsiveness							
Very good/good rating of the last physician’s visit (GP or SP) (vs. moderate/bad/very bad)							
Waiting time until the appointment (*n* = 2611)	2177	83.4	**936**	**77.5 [75.1–79.8]**	**1241**	**88.5 [86.7–90.0]**	**<0.001**
Waiting time in medical practice (*n* = 2643)	1969	74.5	**844**	**68.7 [66.0–71.2]**	**1125**	**79.6 [77.4–81.6]**	**<0.001**
Free choice of physician/practice (*n* = 2607)	2405	92.3	**1066**	**88.3 [86.4–90.0]**	**1339**	**95.6 [94.5–96.6]**	**<0.001**
Respectful treatment (*n* = 2643)	2478	93.8	**1121**	**91.2 [89.5–92.7]**	**1357**	**96.0 [94.8–96.9]**	**<0.001**
Comprehensible explanations (*n* = 2642)	2376	89.9	**1029**	**83.7 [81.6–85.7]**	**1347**	**95.3 [94.1–96.3]**	**<0.001**
Participation in shared decision-making (*n* = 2615)	2262	86.5	**949**	**78.4 [76.0–80.7]**	**1313**	**93.5 [92.1–94.7]**	**<0.001**
Talk confidentially (*n* = 2616)	2452	93.7	**1102**	**90.3 [88.6–91.9]**	**1350**	**96.7 [95.7–97.5]**	**<0.001**
Coordination of care among different physicians (*n* = 1953)	1162	59.5	**418**	**46.1 [42.9–49.3]**	**744**	**71.1 [68.3–73.8]**	**<0.001**
Trust that treatment solely serves for well-being (and not other interests) (*n* = 2602)	2103	80.8	**880**	**73.4 [70.8–75.8]**	**1223**	**87.2 [85.3–88.8]**	**<0.001**
Discrimination experiences in health care in the past year ^a^ (*n* = 2675)	220	8.2	**156**	**12.6 [10.8–14.5]**	**64**	**4.5 [3.5–5.6]**	**<0.001**
High need for reforms in Germany (vs. low/no need)							
Coordination between physicians and hospitals (*n* = 2405)	1153	47.9	**613**	**55.0 [52.0–57.9]**	**540**	**41.9 [39.2–44.6]**	**<0.001**
Coordination between physicians (*n* = 2557)	1217	47.6	**665**	**55.1 [52.3–57.9]**	**552**	**40.9 [38.3–43.5]**	**<0.001**
Amount of money spent out-of-pocket (*n* = 2536)	975	38.4	**511**	**43.5 [40.7–46.4]**	**464**	**34.1 [31.6–36.6]**	**<0.001**
Availability of SPs (*n* = 2762)	641	23.2	**375**	**29.3 [26.9–31.9]**	**266**	**17.9 [16.0–19.9]**	**<0.001**
Quality of care (*n* = 2669)	584	21.9	**323**	**26.1 [23.7–28.6]**	**261**	**18.2 [16.3–20.3]**	**<0.001**
Availability of home care services (*n* = 1877)	413	22.0	**238**	**27.9 [24.9–30.9]**	**175**	**17.1 [14.9–19.5]**	**<0.001**
Availability of GPs (*n* = 2728)	571	20.9	**311**	**24.6 [22.3–27.0]**	**260**	**17.8 [15.9–19.8]**	**<0.001**
Availability of hospitals (*n* = 2745)	168	6.1	**99**	**7.8 [6.4–9.4]**	**69**	**4.7 [3.7–5.8]**	**0.001**
Social and financial risk protection							
Households with out-of-pocket spending ≥500€ in the past year (vs. <500€) (*n* = 2312)	789	34.1	398	36.1 [33.3–39.0]	391	32.3 [29.7–35.0]	0.058
Very strong/strong financial burden by out-of-pocket spending (vs. fair/less strong/not at all) (*n* = 2348)	273	11.6	**168**	**15.1 [13** **.0–** **17.2]**	**105**	**8.5 [7.1–10.2]**	**<0.001**
Difficulties paying health insurance premium ^a^ (*n* = 2660)	73	2.7	**44**	**3.6 [2.7–4.7]**	**29**	**2.0 [1.4–2.8]**	**0.013**
Improved efficiency							
Experiences in the past two years ^a^							
Duplicate tests due to lack of co-ordination (*n* = 2462)	572	23.2	**323**	**28.7 [26.1–31.4]**	**249**	**18.6 [16.6–20.8]**	**<0.001**
Subjectively unnecessary services (e.g., pharmaceuticals) (*n* = 2432)	624	25.7	**358**	**32.4 [29.7–35.2]**	**266**	**20.1 [18.0–22.3]**	**<0.001**
Relation of health insurance premium to coverage (*n* = 2751)							**0.004**
Low/too low	119	4.3	**65**	**5.1 [4.0–6.4]**	**54**	**3.6 [2.8–4.7]**	
Fair	1716	62.4	**753**	**59.2 [56.5–61.9]**	**963**	**65.1 [62.6–67.5]**	
Too high/high	916	33.3	**453**	**35.6 [33.0–38.3]**	**463**	**31.3 [29.0–33.7]**	

Abbreviations: GP—general practitioner; SP—specialist; VAS—visual analogue scale; CI—confidence interval. Notes: ^a^ yes (vs. no); ^b^ multiple answers possible; ^c^ Mann-Whitney U test was used for VAS; bold values indicate a statistically significant difference with a *p*-value < 0.05.

## Data Availability

The data presented in this study are available on request from the corresponding author. The data are not publicly available due to privacy restrictions.
